# Curcumin targets YAP1 to enhance mitochondrial function and autophagy, protecting against UVB-induced photodamage

**DOI:** 10.3389/fimmu.2025.1566287

**Published:** 2025-03-25

**Authors:** Quan Chen, Wenxin Lin, Yi Tang, Fengmei He, Bihua Liang, Jiaoquan Chen, Huaping Li, Huilan Zhu

**Affiliations:** ^1^ Department of Dermatology, Guangzhou Dermatology Hospital, Guangzhou, Guangdong, China; ^2^ Department of Dermatology, Guangzhou First People’s Hospital, Guangzhou, Guangdong, China

**Keywords:** curcumin, UVB, skin damage, YAP1, mitochondrial, autophagy, photoprotection

## Abstract

**Background:**

Ultraviolet B (UVB) radiation is a major environmental factor contributing to skin damage via DNA damage, oxidative stress, inflammation, and collagen degradation. It penetrates the epidermis, disrupts DNA integrity, and generates reactive oxygen species (ROS), activating pro-inflammatory pathways such as NF-κB and AP-1, and inducing matrix metalloproteinases (MMPs). These processes lead to structural skin changes, inflammation, and pigmentation disorders like melasma. Cumulative DNA damage from UVB also drives photocarcinogenesis, with nearly 90% of melanomas associated with UV radiation (UVR). Despite clinical interventions like phototherapy and antioxidants, effective treatments for UVB-induced damage remain limited due to side effects and efficacy issues.

**Methods:**

This study investigates the protective effects of curcumin on UVB-induced skin damage using a mouse UVB irradiation model and HaCaT cells exposed to UVB *in vitro*. Skin damage was assessed through histopathological and immunohistochemical analyses. Cellular functional changes were evaluated using assays for cell viability, mitochondrial function, ROS levels, and apoptosis. Transcriptomic analysis was employed to elucidate the molecular mechanisms underlying curcumin’s protective effects on HaCaT cells post-UVB exposure. This integrated approach provides a comprehensive understanding of curcumin’s molecular-level protection against UVB-induced skin damage.

**Results:**

Curcumin significantly alleviated UVB-induced skin lesions and inflammation *in vivo*. *In vitro*, it mitigated UVB-induced HaCaT cell damage, enhancing viability while reducing apoptosis and ROS levels. Transcriptomic analysis revealed that curcumin upregulated YAP signaling and mitochondrial autophagy while suppressing IL-18 expression.

**Conclusion:**

Curcumin treatment markedly improved UVB-induced skin lesions and reduced epidermal inflammation and thickness *in vivo*. *In vitro*, curcumin intervention alleviated UVB-induced HaCaT cell damage, including reduced viability, increased apoptosis, elevated ROS and DNA damage, and enhanced inflammatory responses. Transcriptomic analysis demonstrated that curcumin upregulated the YAP signaling pathway and mitochondrial autophagy while inhibiting the IL-18 pathway. Further studies revealed that curcumin directly interacts with YAP1, promoting mitochondrial autophagy, an effect blocked by the YAP1 inhibitor Verteporfin. Additionally, curcumin enhances mitochondrial function through YAP1, maintaining mitochondrial integrity and preventing the release of mitochondrial DNA (mtDNA) and mitochondrial ROS (mtROS), thereby suppressing NLRP3/IL-18 pathway activation.

## 1 Introduction

Ultraviolet B (UVB) radiation (280-320 nm) is a major environmental factor causing skin damage through DNA damage, oxidative stress, inflammation, and collagen alterations (1–4). UVB penetrates the epidermis, disrupting DNA structure and generating reactive oxygen species (ROS), which activate pro-inflammatory pathways (e.g., NF-κB and AP-1) and induce matrix metalloproteinases (MMPs) (5–11). This leads to skin thickening, hyperplasia, inflammation, pigmentation disorders (e.g., melasma), and photocarcinogenesis, with nearly 90% of melanomas linked to UVB exposure (12–16).

Current clinical measures for UVB-induced photodamage include antioxidants (e.g., vitamin E), sunscreens, and protective clothing. However, these methods face challenges such as limited stability, bioavailability, and potential side effects. Therefore, identifying more effective treatments is crucial (17–19). Natural extracts with unique biological activities and low toxicity are considered promising therapeutic candidates (20–23). For instance, theaflavins repair cellular structures and reduce UVB-induced apoptosis in HaCaT cells (24, 25). Licorice, Rhodiola, and Astragalus extracts inhibit skin cell apoptosis by reducing TNF-α levels (26–28). Resveratrol combats skin aging by enhancing antioxidant enzyme activity (29, 30). However, these extracts often suffer from low extraction efficiency and incomplete mechanistic understanding.

Curcumin, a lipophilic polyphenolic compound extracted from turmeric rhizomes, has demonstrated multiple biological activities, including antioxidant, anti-inflammatory, promoting DNA repair, and inhibiting collagen degradation (31–36). In addition, its low toxicity and high safety make it suitable for long-term use, surpassing some chemically synthesized drugs. Topical curcumin application has been shown to inhibit UVB-induced inflammation and collagen disorder. However, the specific mechanisms underlying curcumin’s protective effects against skin photodamage remain unclear. Therefore, a deep understanding of the anti-inflammatory and antioxidant mechanisms of curcumin will provide a solid theoretical basis for developing treatment strategies for UVB damage and related diseases based on curcumin.

**Figure 1:**
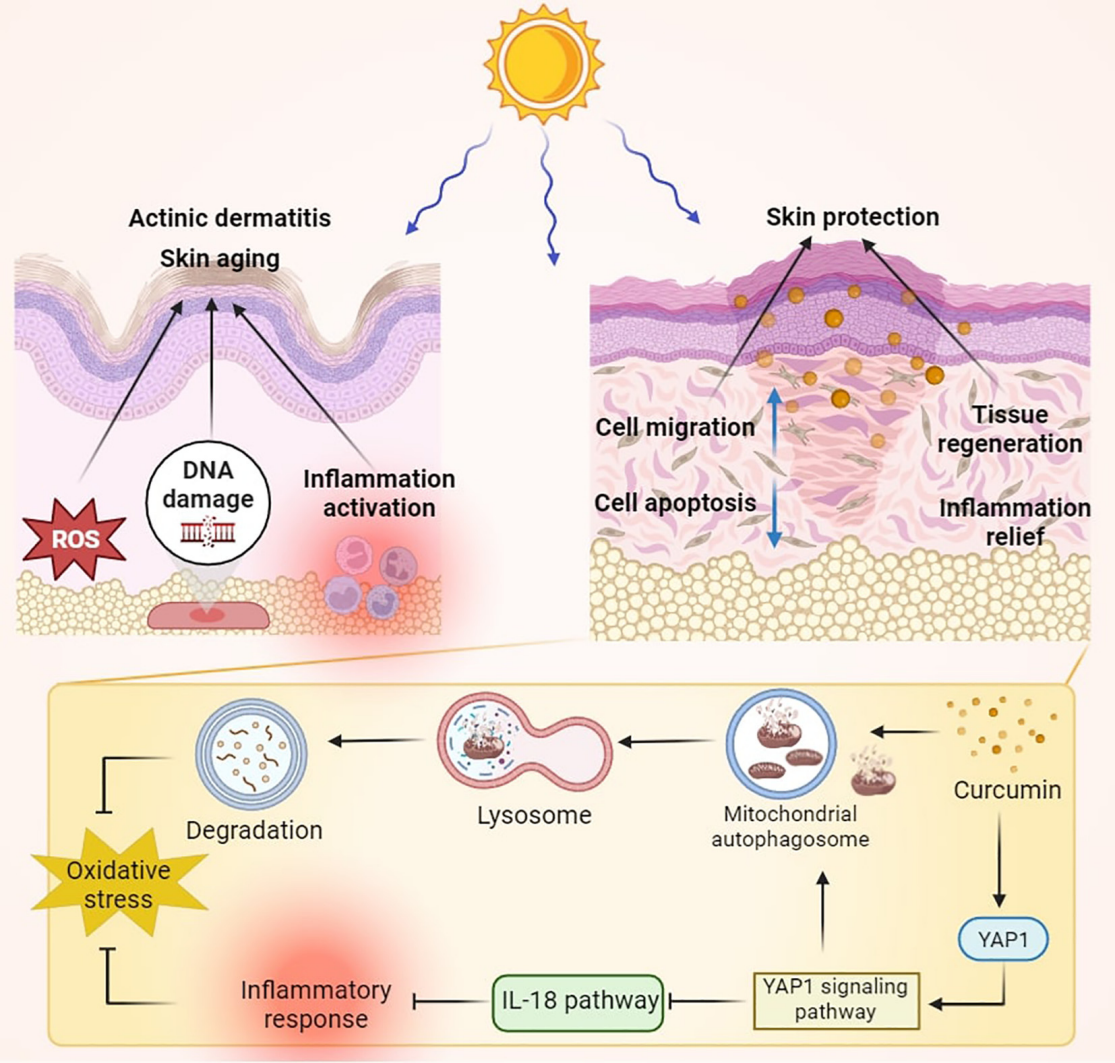
Schematic diagram of curcumin treatment improving skin damage caused by UVB irradiation. Curcumin intervention significantly enhanced the YAP signaling pathway, led to increased mitochondrial autophagy, and inhibited the IL-18 pathway, thereby combating UVB-induced oxidative and inflammatory damage.

This study aims to investigate the protective effects of curcumin on skin tissue and keratinocyte damage caused by UVB radiation using a mouse UVB irradiation model and HaCaT cells exposed to UVB *in vitro*. In this study, we first clarified the skin damage caused by UVB irradiation through pathological and immunohistochemical analyses of tissue samples. Subsequently, we systematically analyzed changes in cellular functions using methods such as cell viability, mitochondrial function analysis, ROS analysis, and apoptosis analysis. Furthermore, we employed transcriptomic analysis to elucidate the molecular basis of curcumin’s protective effects on HaCaT cells post-UVB intervention and to further clarify its mechanism of action. Our findings demonstrate that curcumin administration notably ameliorated skin damage induced by UVB exposure, as well as reduced inflammation and altered thickness within the epidermis *in vivo*. *In vitro* studies utilizing HaCaT cells indicated that following UVB treatment, there was a decline in cell viability, impeded cell migration, heightened apoptosis, a marked increase in ROS and DNA damage, and an exacerbation of inflammatory responses. Curcumin treatment, when compared to the UVB-only group, substantially reduced cellular impairment. Transcriptomic profiling identified that curcumin significantly bolstered the YAP signaling pathway and mitochondrial autophagy, while concurrently inhibiting the IL-18 pathway. Further investigation disclosed that curcumin interacts directly with YAP1, augmenting mitochondrial autophagy, a process that can be inhibited by the YAP1 antagonist Verteporfin. Collectively, curcumin interacts with YAP1 to modulate mitochondrial autophagy and provides protective effects. This research not only expands our comprehension of curcumin’s mechanisms but also furnishes robust scientific validation for its use in countering UVB-induced oxidative stress and inflammation (**Figure 1**).

## 2 Materials and methods

### 2.1 Animal experiments

A mouse model of UVB-induced skin damage was established, and mice were randomly divided into five experimental groups, including Ctrl, UVB (100 mJ/cm^2^, 30 min), UVB+0.05% DMSO, UVB+Cur-L (5 micromolar (µM)), and UVB+Cur-H (10 µM). At the end of the experiment, mouse back skin samples were collected, and photographs of the mouse back skin were taken for preliminary assessment of skin damage severity.

The collected skin samples were stained with H&E to further assess the severity of skin damage.

The thickness of the skin epidermis was measured using H&E-stained sections to quantify UVB-induced skin damage. Inflammatory cell infiltration was observed through H&E staining to assess the inflammatory response.

Masson staining was performed on the samples to assess the distribution and content of collagen fibers.

Proteins extracted from skin tissue were used to detect the levels of IL-18, IL-1β, and TNF-α by ELISA to assess the expression of inflammatory factors.

Statistical analysis was performed on all experimental data to compare differences between the experimental groups and to evaluate the protective effect of curcumin.

### 2.2 Cell culture

HaCaT cells were cultured in MEM (Dulbecco’s Modified Eagle Medium) supplemented with 10% Fetal Bovine Serum (FBS) and 1% Penicillin-Streptomycin (P/S). The cells were maintained in an incubator with a gas mixture of 95% air and 5% carbon dioxide (CO_2_) at a temperature of 37°C. Subculturing was performed when the cell confluence reached 80% to ensure optimal cell growth.

### 2.3 CCK-8

HaCaT cells were seeded in a 96-well plate and cultured for 24 h. Subsequently, various treatment groups were co-incubated with the cells, including the control (Ctrl), UVB (500 mJ/cm^2^, 15 min), UVB+0.05% DMSO, UVB+low-concentration curcumin (UVB+Cur-L), and UVB+high-concentration curcumin (UVB+Cur-H). Finally, cell viability was assessed using the standard CCK-8 assay. Absorbance in each well at 450 nm was recorded using a microplate reader, and cell viability was calculated using the following equation:


$$\text{Cell viability }\left(\%\right)=\left({\text{OD}}_{\text{treat}}-{\text{OD}}_{\text{blank}}\right)÷\left({\text{OD}}_{\text{control}}-{\text{OD}}_{\text{blank}}\right)\times 100\%$$

Where OD_treat_ refers to the absorbance of different treatment wells, OD_blank_ is the absorbance of blank wells, and OD_control_ means the absorbance of wells without any treatment.

### 2.4 Scratch assay

Initially, HaCaT cells were seeded into a 6-well plate and allowed to grow to 80-90% confluence. Subsequently, three parallel lines were marked on the back of the 6-well plate as reference guides using a marker and ruler, and straight lines were drawn across the cell layer with a 200μL pipette tip to create multiple fixed observation points. After the scratch was made, the cells were gently rinsed with PBS buffer 2 to 3 times to remove non-adherent cells. The cells were then subjected to various treatments. The control group (Ctrl) received no treatment, the UVB group was exposed to UVB radiation (50 mJ/cm^2^, 15 min), the UVB+0.05% DMSO group was supplemented with 0.05% DMSO, and the UVB+Cur-L and UVB+Cur-H groups were treated with low and high concentrations of curcumin, respectively. At the 0-h mark, images of the scratch area were captured using a microscope to record the initial wound width. Thereafter, the cells were placed in an incubator for culture, and the cell migration into the scratch area was observed and photographed at different time points (0 h, 24 h). Finally, the changes in wound width over time were measured using image processing software or manual measurement to assess the effect of each experimental group on the migration ability of HaCaT cells.

### 2.5 ROS scavenging

HaCaT cells were incubated with 1 mL of DCFH-DA solution, which was diluted in FBS-free DMEM medium at a ratio of 1:1000. After a 20-minute incubation, the cells were subjected to various treatments, including Ctrl, UVB (50 mJ/cm^2^, 15 min), UVB+0.05% DMSO, UVB+Cur-L, and UVB+Cur-H. Untreated cells served as the control group. Following a 1-h incubation, fluorescence intensity was promptly recorded using confocal laser scanning microscopy (CLSM).

### 2.6 Mitochondrial membrane potential assessment via JC-1staining

The effects of UVB radiation and various treatments on mitochondrial membrane potential were assessed using JC-1 staining. HaCaT cells were cultured in dishes or plates to an appropriate confluence (typically 70-90%). The cells were then subjected to different treatments, including Ctrl, UVB (50 mJ/cm^2^, 15 min), UVB+0.05% DMSO, UVB+Cur-L, and UVB+Cur-H. After 12 h, cells were washed with PBS to remove unbound drugs or medium. JC-1 staining was performed on the different groups. JC-1 dye was added to the cell culture medium at a concentration of 2 micromolar (μM) and incubated in the incubator for 20-30 min. After staining, cells were washed with PBS to remove unbound JC-1 dye. The distribution and intensity of red and green fluorescence were observed and recorded using a confocal laser scanning microscope.

### 2.7 Animal ethics

C57 mice were sourced from the Shanghai Model Organisms Center. These mice were maintained in a controlled environment with a temperature range of 20-30°C (± 5°C), relative humidity of 50%-70% (± 10%), and a 12-hour light-dark cycle. Throughout the study, the animals had unrestricted access to water and food. All animal procedures were conducted in strict compliance with the guidelines established by the Institutional Animal Care and Use Committee (IACUC) of Shanghai Tenth Peoples Hospital, under approval number SHDSYY-2024-19846.

### 2.8 Statistical analysis

The experimental data were expressed as the mean ± standard deviation (SD). Each quantitative assessment was replicated at least three times to ensure reliability. Statistical significance between two groups was determined using a two-tailed Student’s t-test. For comparisons involving more than two groups, One-way analysis of variance (ANOVA) with Tukey multiple comparison test for multiple comparisons was employed. All statistical analyses were conducted using GraphPad Prism 9.0 software. The results were considered statistically significant at the following levels: #### *P <*0.0001, ### *P* < 0.001, **** P <0.0001, *** *P <*0.001, ** *P <*0.01, * *P*<0.05, and ns *P >*0.05, as indicated by the respective symbols.

## 3 Result

### 3.1 The therapeutic effect of curcumin on skin damage caused by UVB irradiation

**Figure 2:**
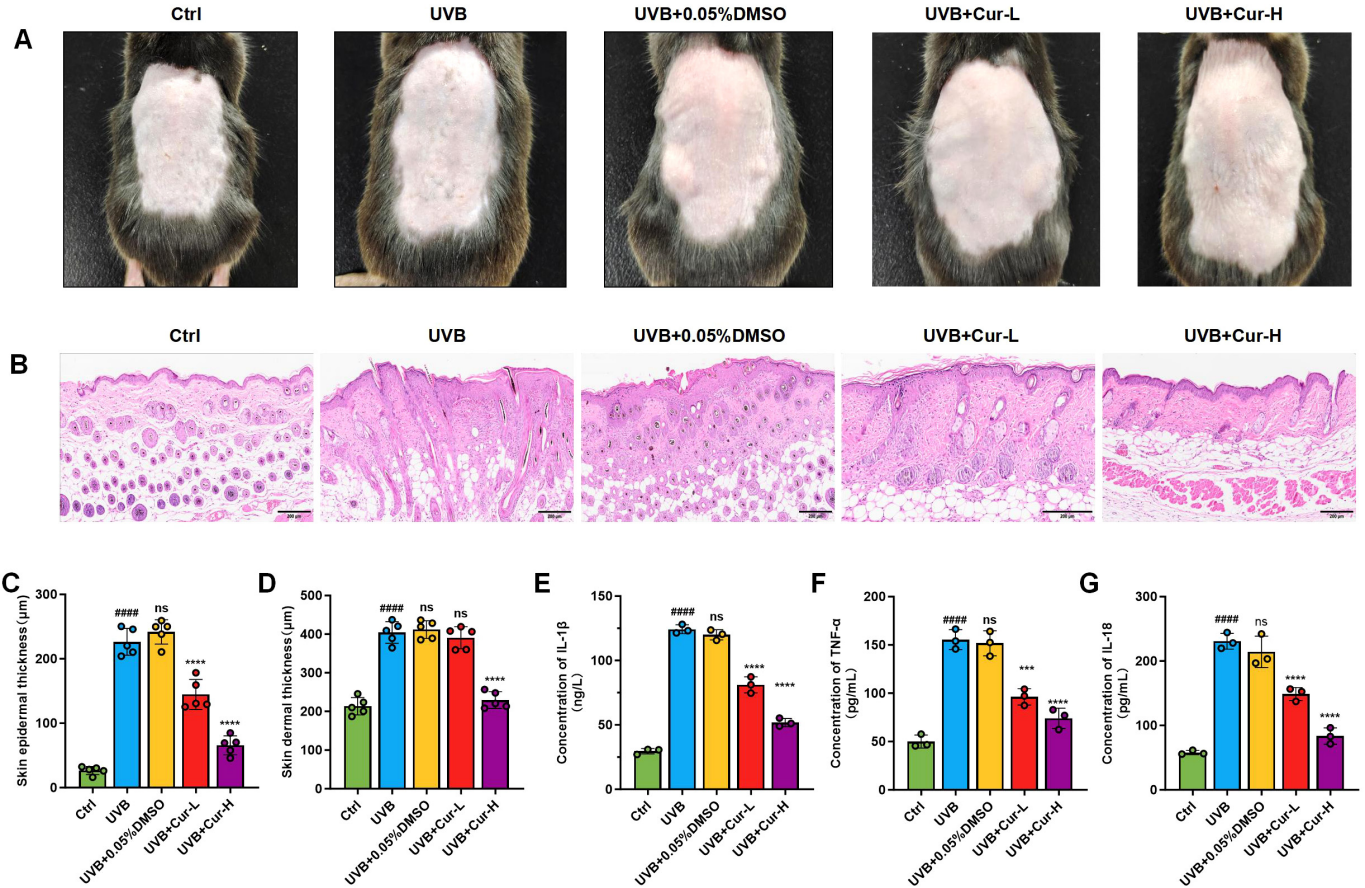
The mitigation of UVB-induced skin damage of curcumin. **(A)** Optical photos of mouse skin after different treatments, including Ctrl, UVB, UVB+0.05%DMSO, UVB+Cur-L, and UVB+Cur-H. **(B)** H&E staining results of mouse skin sections after different treatments, including Ctrl, UVB, UVB+0.05%DMSO, UVB+Cur-L, and UVB+Cur-H. **(C, D)** Quantitative results of skin epidermal thickness and skin dermal thickness of mouse skin sections after different treatments, including Ctrl, UVB, UVB+0.05%DMSO, UVB+Cur-L, and UVB+Cur-H (n=5). **(E–G)** The inflammatory cytokines level (IL-1β, TNF-α, and IL-18) of skin tissues after different treatments, including Ctrl, UVB, UVB+0.05%DMSO, UVB+Cur-L, and UVB+Cur-H (n=5). The data are presented as mean value ± SD. Statistical significance was calculated using one-way analysis of variance (ANOVA) with Tukey multiple comparison. #### *P <*0.0001 vs Ctrl, ns *P >*0.05 vs UVB, *** *P* <0.001 vs UVB, **** *P* <0.0001 vs UVB.

We initially established a UVB-induced skin damage model to assess the therapeutic effects of curcumin on skin damage in mice. After different treatments, skin tissue was collected for histological evaluation. By directly observing the dorsal skin damage in mice, we noted that the UVB-treated group exhibited severe skin damage, with a severity score of 10. In contrast, the UVB+0.05% DMSO group did not demonstrate protective effects, also receiving a severity score of 10. Mice in the UVB+Cur-L and UVB+Cur-H groups showed a significant reduction in skin damage, with severity scores decreasing to 5 and 2, respectively (Fiure 2A). Hematoxylin and eosin (H&E) staining revealed that the UVB-treated group had disrupted skin tissue structure and marked inflammatory cell infiltration. The UVB+Cur-L and UVB+Cur-H groups, however, showed improvements in tissue structure and reductions in inflammatory cell infiltration (**Figure 2B**). Measurements of epidermal thickness and dermal thickness were consistent with these observations. The UVB-treated group had a significant increase in epidermal thickness, while curcumin-treated groups showed a significant decrease, particularly in the high-concentration curcumin group, approaching the levels seen in the Ctrl group (**Figures 2C, D**). Finally, we utilized enzyme-linked immunosorbent assay (ELISA) kit to detect the expression levels of inflammatory cytokines IL-1β, TNF-α, and IL-18 in mouse skin tissue after different treatments. As shown in **Figures 2E–G**, the UVB-treated group had significantly elevated levels of inflammatory cytokines, while the UVB+Cur-H group had significantly reduced levels.

### 3.2 UVB-induced cell damage alleviation of curcumin *in vitro*

**Figure 3:**
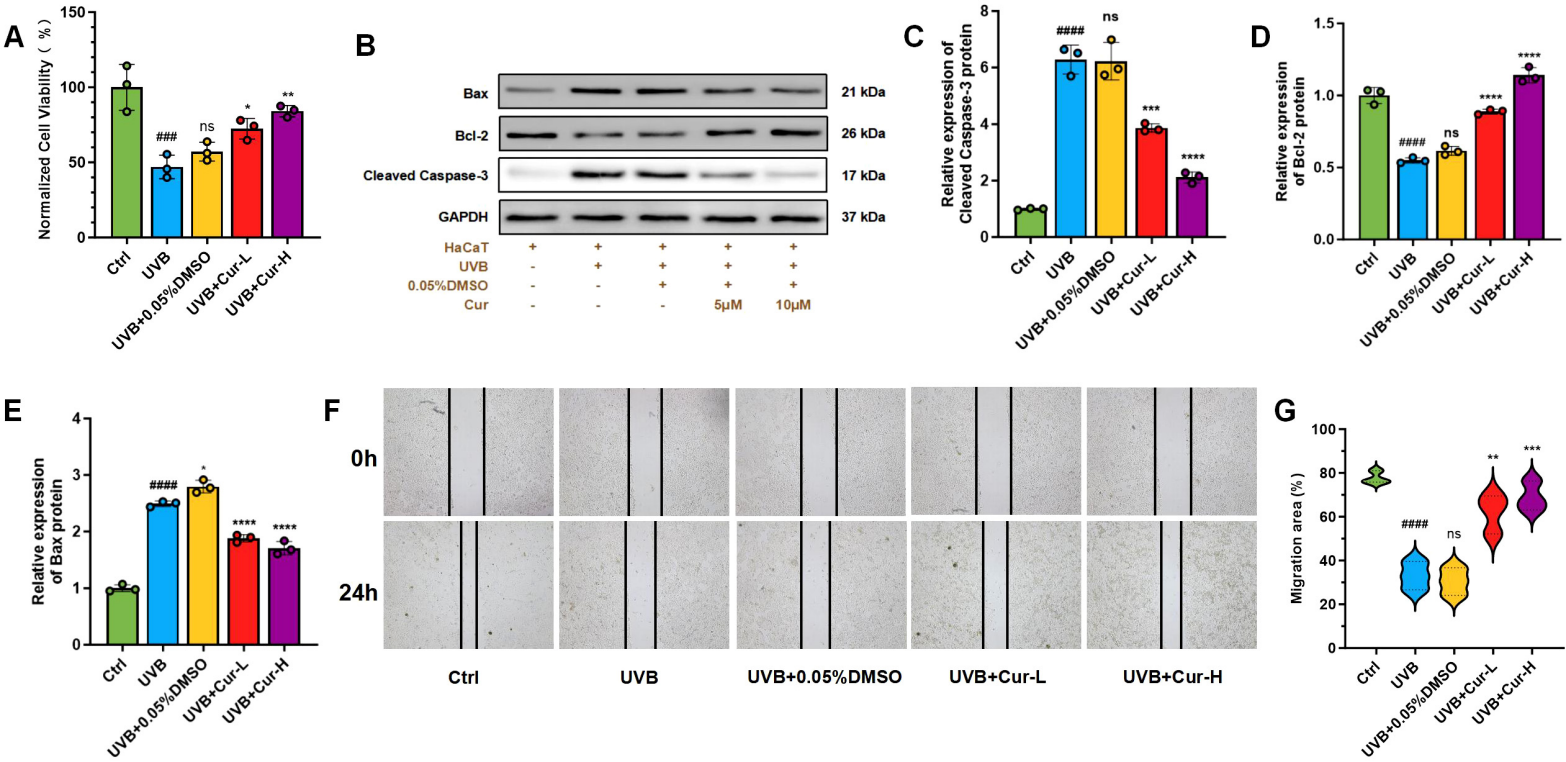
UVB-induced cell damage alleviation of curcumin *in vitro*. **(A)** Cell viability of HaCaT cells after different treatments, including Ctrl, UVB, UVB+0.05%DMSO, UVB+Cur-L, and UVB+Cur-H (n=3). **(B)** Western blot analysis for apoptotic markers, including Bax, Bcl-2, and Cleaved Caspase-3. **(C–E)** The relative expression of Cleaved Caspase-3, Bcl-2, and Bax of cells after different treatments (n=3). **(F)** The progression of cell migration in HaCaT cells at 0 and 24 h after different treatments, including Ctrl, UVB, UVB+0.05%DMSO, UVB+Cur-L, and UVB+Cur-H. **(G)** Quantitative analysis results of cell scratch assay from **(B)** (n=3). The data are presented as mean value ± SD. Statistical significance was calculated using one-way analysis of variance (ANOVA) with Tukey multiple comparison. ### *P* < 0.001 vs Ctrl, #### *P* < 0.0001 vs Ctrl, ns *P* > 0.05 vs UVB, * *P*<0.05 vs UVB, ** *P <*0.01 vs UVB, *** *P <*0.001 vs UVB, **** *P* < 0.0001 vs UVB.

UVB radiation induces oxidative stress and free radical damage, alters microRNA expression, causes DNA damage, affects cell cycle and autophagosome formation, induces premature cell aging, and reduces cell migration ability, leading to a decrease in cell viability. These factors interact with each other, collectively leading to a decrease in cellular physiological function and proliferation ability (37, 38). Experimental results showed that compared with the control group, the cell viability of HaCaT cells treated with UVB radiation decreased to 47.5% (**Figure 3A**). The cell viability of the UVB+0.05%DMSO treatment group also decreased to 57.3%. Curcumin, with its antioxidant, anti-inflammatory, and regulation of cell signaling properties, can reduce oxidative stress damage to cells and demonstrate the potential to protect cells from UVB radiation damage (39, 40). As shown in **Figure 3A**, after treatment with 5 µM low concentration curcumin (UVB+Cur-L), the cell damage caused by UVB was effectively alleviated, and the cell viability increased to 72.6%. After treatment with 10 µM high concentration curcumin (UVB+Cur-H), cell viability was significantly restored to 84.8%.

Western blot (WB) analysis revealed that UVB radiation significantly increased the expression of pro-apoptotic protein Bax and Cleaved Caspase-3, while concurrently decreasing the expression of the anti-apoptotic protein Bcl-2. However, treatment with curcumin, particularly at high concentrations, significantly suppressed the UVB-induced upregulation of Bax and activation of Caspase-3, and enhanced the expression of Bcl-2, thereby exerting an anti-apoptotic effect (**Figures 3B–E**).

Scratch assays were further utilized to assess the reparative effects of curcumin on UVB-induced impairment of cell migration (**Figures 3F, G**). After 12 h of various treatments, compared to the control group, UVB irradiation significantly reduced the cell migration rate to 30.5%, indicating that UVB radiation may inhibit the migratory ability of HaCaT cells. Treatment with UVB plus 0.05% DMSO reduced the cell migration rate to 36.3%. Treatment with low-dose curcumin gradually restored the cell migration rate to 56.7%. More notably, treatment with high-dose curcumin increased the cell migration rate to 68.5%.

### 3.3 UVB-induced oxidative stress damage reduction of curcumin *in vitro*

**Figure 4:**
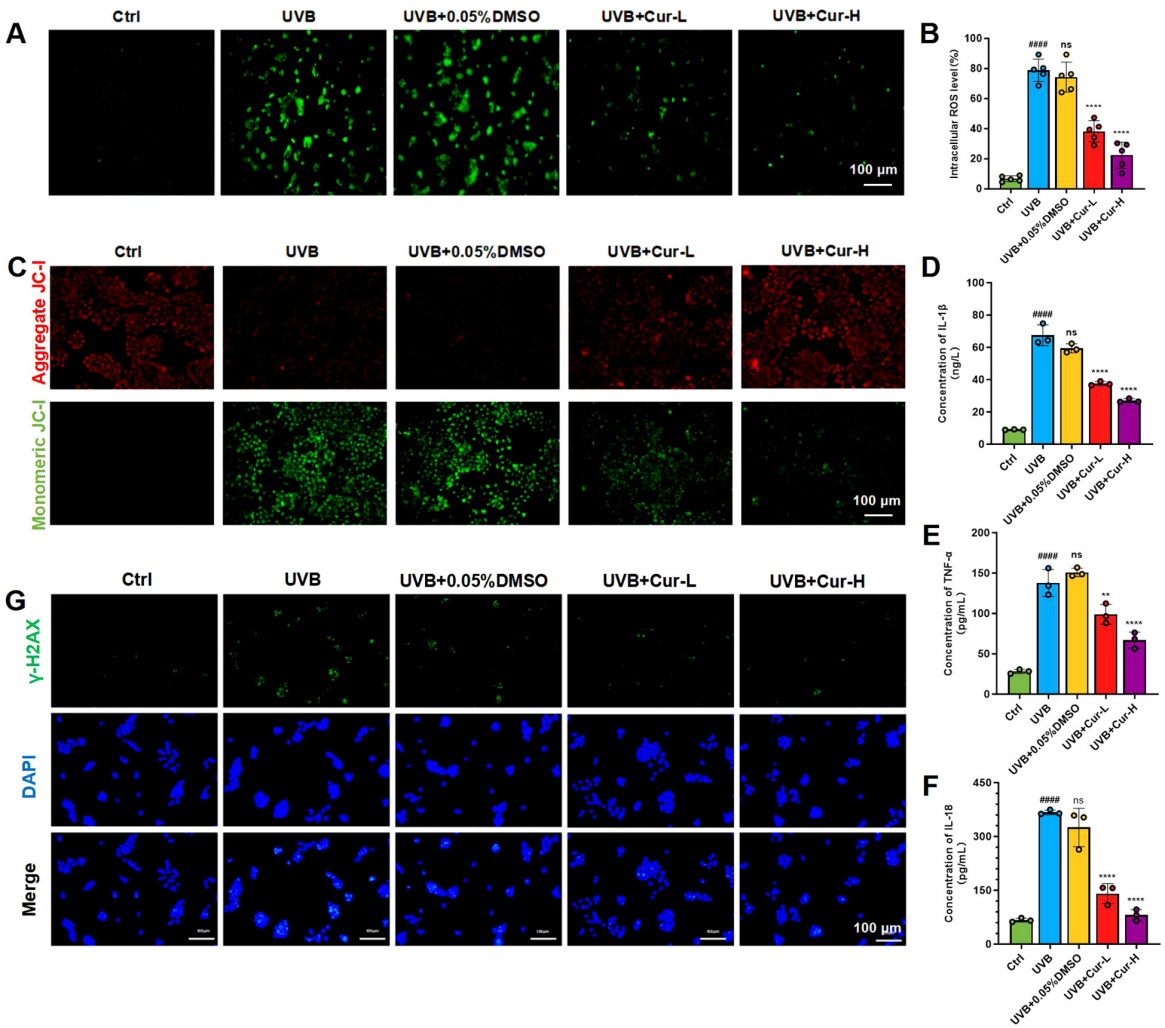
UVB-induced oxidative stress damage reduction of curcumin *in vitro*. **(A)** CLSM images of HaCaT cells stained with DCFH-DA probe after different treatments, including Ctrl, UVB, UVB+0.05%DMSO, UVB+Cur-L, and UVB+Cur-H. **(B)** Quantification of CLSM images results (n=5). **(C)** JC-1 staining of HaCaT cells after different treatments for MMP assessment. **(D–F)** The cytokines level (IL-1β, TNF-α, and IL-18) of HaCaT cells after different treatments, including Ctrl, UVB, UVB+0.05%DMSO, UVB+Cur-L, and UVB+Cur-H (n=3). **(G)** γ-H2AX and DAPI staining for DNA damage evaluation of HaCaT cells after different treatments, including Ctrl, UVB, UVB+0.05%DMSO, UVB+Cur-L, and UVB+Cur-H. The data are presented as mean value ± SD. Statistical significance was calculated using one-way analysis of variance (ANOVA) with Tukey multiple comparison. ### *P* < 0.001 vs Ctrl, #### *P <*0.0001 vs Ctrl, ns *P* > 0.05 vs UVB, ** *P* < 0.01 vs UVB, **** *P* < 0.0001 vs UVB.

UVB photons can be directly absorbed by DNA, lipids, and proteins inside cells, triggering the formation of electron excited states and generating singlet oxygen and other ROS (41, 42). We further employed the 2’,7’-Dichlorodihydrofluorescein Diacetate (DCFH-DA) assay to measure the levels of ROS in HaCaT cells, thereby assessing the damaging effects of UVB radiation on skin cells and the potential protective role of curcumin. Upon oxidation by ROS within the cells, DCFH-DA is converted into DCF, which emits a strong green fluorescence. The experimental results demonstrated that UVB radiation significantly increased the intracellular DCF fluorescence intensity, indicating a substantial induction of ROS production within the cells by UVB. Compared to the UVB-only group, the DCF fluorescence intensity in the UVB+0.05% DMSO group showed no significant change, suggesting that DMSO as a solvent control has minimal impact on ROS levels. After treatment with curcumin, there was a significant reduction in the green fluorescence intensity within the cells, particularly in the UVB+Cur-H group (**Figures 4A, B**). The JC-1 dye, a lipophilic cationic dye, exhibits fluorescence properties that vary with changes in the mitochondrial membrane potential. Under normal conditions, due to the high mitochondrial membrane potential, cells predominantly display red fluorescence. When the mitochondrial membrane potential decreases, the red fluorescence diminishes while the green fluorescence intensifies. Utilizing JC-1 dye to monitor changes in the mitochondrial membrane potential (MMP) of HaCaT cells, the results indicated that UVB radiation led to a decrease in MMP, reflected by an increase in green fluorescence and a decrease in red fluorescence. After treatment with curcumin, there was a significant reduction in green fluorescence within the cells (**Figure 4C**).

ELISA kits were utilized to detect the expression levels of inflammatory cytokines IL-1β, TNF-α, and IL-18 in the supernatants of HaCaT cell cultures, thereby assessing the impact of UVB radiation on inflammatory responses and the potential anti-inflammatory effects of curcumin. The experimental results shown that UVB radiation significantly elevated the expression levels of these inflammatory cytokines. In contrast, the groups treated with UVB+Cur-L and UVB+Cur-H exhibited reduced expression levels of the cytokines (**Figures 4D–F**). Immunofluorescence assays were further employed to detect the expression levels of 8-hydroxy-2’-deoxyguanosine (8-OHdG) in HaCaT cells, thereby assessing the impact of UVB radiation on DNA oxidative damage and the potential protective effects of curcumin. 8-OHdG, a significant biomarker of DNA oxidative damage, indicates exacerbated DNA damage when its expression levels are elevated. The experimental results demonstrated that UVB radiation significantly increased the number of 8-OHdG-positive cells, indicating that UVB can induce DNA oxidative damage. After treatment with UVB+Cur-H, there was a noticeable decrease in the number of 8-OHdG-positive cells (**Figure 4G**).

### 3.4 Molecular docking and RNA-seq

**Figure 5:**
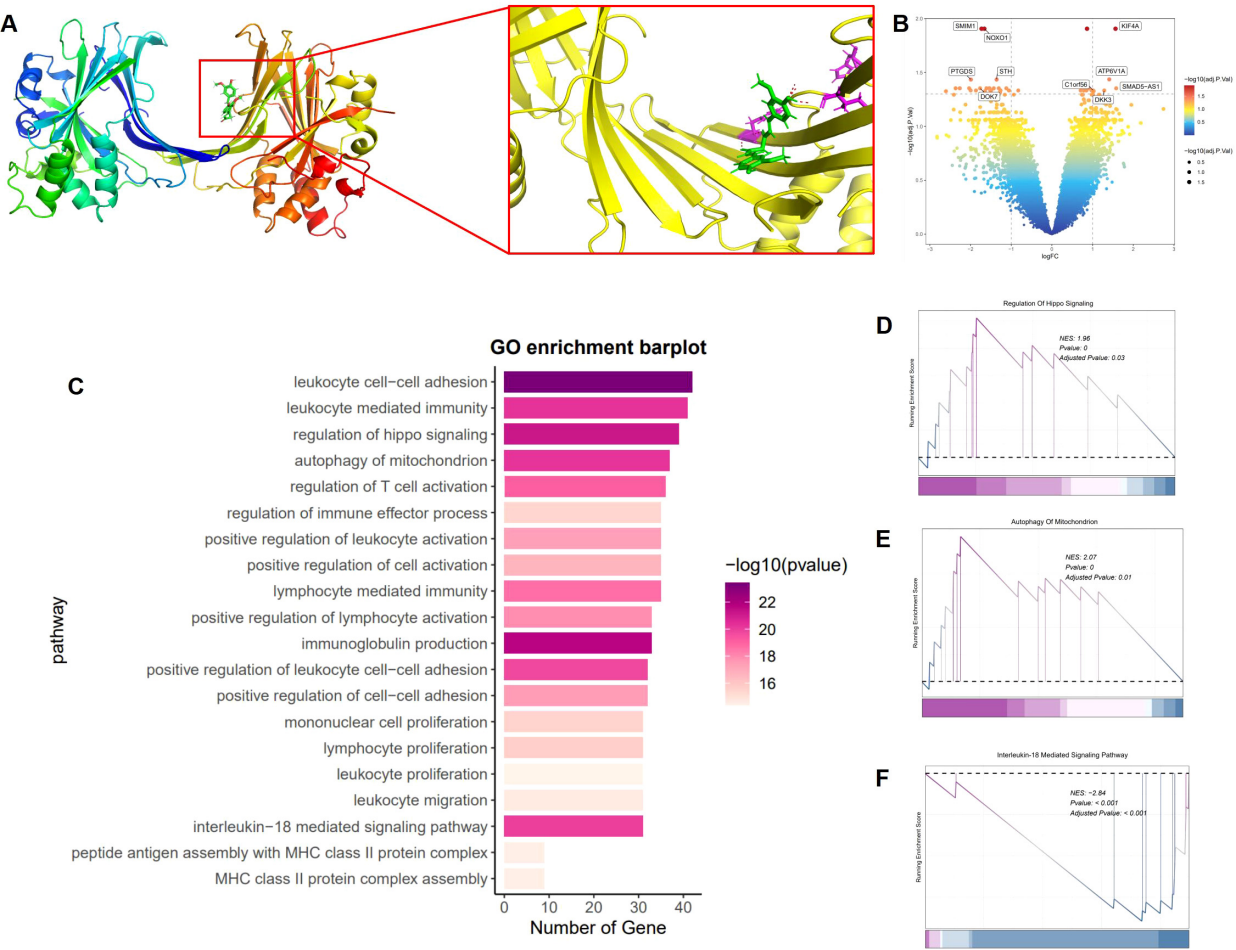
Molecular docking and RNA-seq. **(A)** Molecular interaction network between YAP1 and curcumin. **(B)** Volcano plot of DEGs between UVB and curcumin+UVB groups. (|Fold Change|≥2, p<0.05). **(C)** GO enrichment of DEGs between UVB and curcumin+UVB groups. (|Fold Change|≥2, p<0.05). **(D–F)** GSEA analysis of DEGs between UVB and curcumin+UVB groups. (|Fold Change|≥2, p<0.05).

We investigated the molecular interaction between curcumin and the YAP1 protein to uncover its potential role in treating UVB-induced skin damage. Molecular docking simulations were conducted to calculate the binding energy between curcumin and YAP1, thereby predicting their likely binding modes. The molecular docking analysis revealed stable binding patterns between curcumin and YAP1. Further analysis identified key amino acid residues within YAP1 that interact with curcumin, including Leu351, Asn354, and Val357, which form a stable binding network with curcumin through hydrogen bonds and hydrophobic interactions, thereby influencing YAP1 functionality (**Figure 5A**).

To explore the impact of curcumin on UVB-induced skin damage, we employed RNA sequencing to analyze gene expression profiles. Total RNA extracted from cells post-treatment was subjected to transcriptome analysis, allowing us to compare gene expression differences between curcumin-treated and UVB-exposed groups. We identified differentially expressed genes (DEGs) by intersecting the gene expression profiles. The results indicated significant gene alterations induced by UVB, characterized by the downregulation of SMIM23, NOXO1, STH, PTGDS, and DOK7, and the upregulation of KIF4A, C1orf56, D3K3, SMAD5-AS1, and ATP6V1A (|Fold Change|≥2, p<0.05) (**Figure 5B**). To gain insights into the functions of these DEGs, we performed Gene Ontology (GO) analysis (**Figure 5C**). The GO analysis revealed that these significantly differentially expressed genes were predominantly associated with immune response-related biological processes, such as leukocyte cell-cell adhesion, leukocyte-mediated immunity, regulation of hippo signaling, mitochondrial autophagy, T cell activation, and immune effector process, as well as autophagy-related processes. To visually represent the influence of curcumin on these pathways, we conducted Gene Set Enrichment Analysis (GSEA). The analysis demonstrated a significant positive enrichment of gene expression changes related to the Hippo signaling pathway in the curcumin-treated group compared to the UVB group (**Figure 5D**). Additionally, the expression changes of genes associated with mitochondrial autophagy were more pronounced in the curcumin-treated group (**Figure 5E**). Furthermore, genes related to the IL-18-mediated signaling pathway showed a significant negative enrichment in the curcumin-treated group, indicating a downregulation of these genes (**Figure 5F**).

### 3.5 YAP1 signal pathway regulation of curcumin

**Figure 6:**
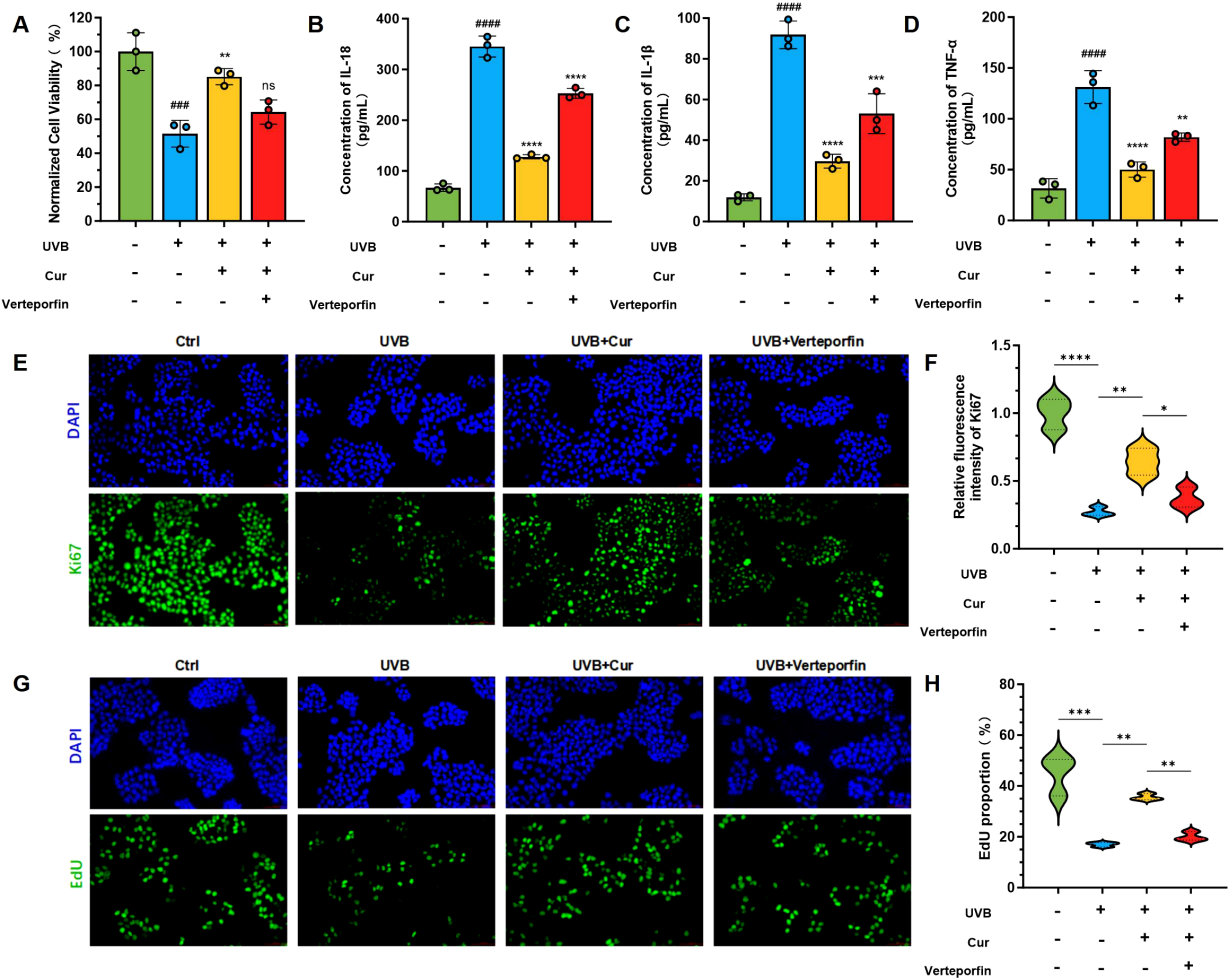
YAP1 signal pathway regulation of curcumin. **(A)** Cell viability of HaCaT cells after different treatments, including Ctrl, UVB, UVB+Cur, and Cur+Verteporfin (n=3). **(B–D)** The cytokines level (IL-1β, TNF-α, and IL-18) of HaCaT cells after different treatments, including Ctrl, UVB, UVB+Cur, and Cur+Verteporfin (n=3). **(E, F)** Immunofluorescence staining for γ-H2AX and the corresponding fluorescence quantitative analysis (n=3). **(G, H)** Immunofluorescence staining for EdU and the corresponding fluorescence quantitative analysis (n=3). The data are presented as mean value ± SD. Statistical significance was calculated using one-way analysis of variance (ANOVA) with Tukey multiple comparison. ### *P* < 0.001 vs Ctrl, #### *P* < 0.0001 vs Ctrl, ns *P* > 0.05 vs UVB, ** *P* < 0.01 vs UVB, *** *P* < 0.001 vs UVB, **** *P* < 0.0001 vs UVB.

To ascertain the direct interaction between curcumin and YAP1, as well as its regulatory effect on mitophagy, we evaluated the expression levels of key proteins involved in the YAP signaling pathway and mitophagy. The CCK-8 assay revealed that UVB radiation significantly diminished cell viability. Curcumin treatment increased cell survival post-UVB exposure, uncovering its protective role against UVB-induced phototoxicity. The addition of the YAP1 inhibitor Verteporfin led to a further decrease in cell viability (**Figure 6A**). We first quantified the secretion levels of inflammatory cytokines in each group using ELISA. Specifically, we measured the concentrations of IL-18, IL-1β, and TNF-α (**Figures 6B–D**). UVB exposure elevated the levels of these inflammatory mediators, which were substantially reduced with curcumin supplementation. However, the co-administration of curcumin and Verteporfin attenuated the curcumin-induced decrease in cytokine levels. To further elucidate the protective mechanisms of curcumin against UVB-induced cellular damage, we assessed the expression of proliferation markers Ki67 and EdU via immunofluorescence staining. Ki67, a marker of cellular proliferation, was reduced upon UVB treatment. The addition of curcumin notably elevated the proportion of Ki67-positive cells (**Figures 6E, F**). However, co-treatment with curcumin and Verteporfin led to a decrease in Ki67-positive cells. EdU, an analog of thymidine used to measure DNA synthesis, indicated a reduction in the rate of cell proliferation following UVB exposure. Curcumin treatment restored this capacity, but its effect was diminished when Verteporfin was introduced (**Figures 6G, H**).

WB analysis and the quantitative protein expression levels results of the Yes-associated protein (YAP) signaling pathway revealed an increase in the phosphorylation of YAP1 (p-YAP1) upon UVB exposure when compared to the control group. This elevation in p-YAP1 signifies an augmented activation of the YAP signaling cascade. In stark contrast, both the UVB+Cur-L and UVB+Cur-H groups demonstrated a reduction in p-YAP1 levels. Furthermore, curcumin can activate the expression of TAZ (Toll-like receptor-associated kinase), a crucial mediator that enhances cellular resistance to apoptosis (**Supplementary Figures S1A–D**). To substantiate the protective effects of curcumin against UVB-induced cellular damage and inflammatory responses, we employed quantitative polymerase chain reaction (q-PCR) to assess the expression levels of pivotal cytokines and proteins. Our findings shown a upregulation of NLRP3 and Caspase-1 mRNA in the UVB group (**Supplementary Figures 2A, B**). Curcumin treatment attenuated the expression of these inflammatory mediators, as evidenced by a substantial decrease in NLRP3 and Caspase-1 mRNA levels. However, the co-administration of Verteporfin, an inhibitor of the YAP1 signaling pathway, partially abrogated these protective effects. Furthermore, the UVB group exhibited elevated levels of IL-1β, TNF-α, and IL-1β mRNA, which were effectively mitigated by curcumin treatment (**Supplementary Figures S3A–C**).

**Figure 7:**
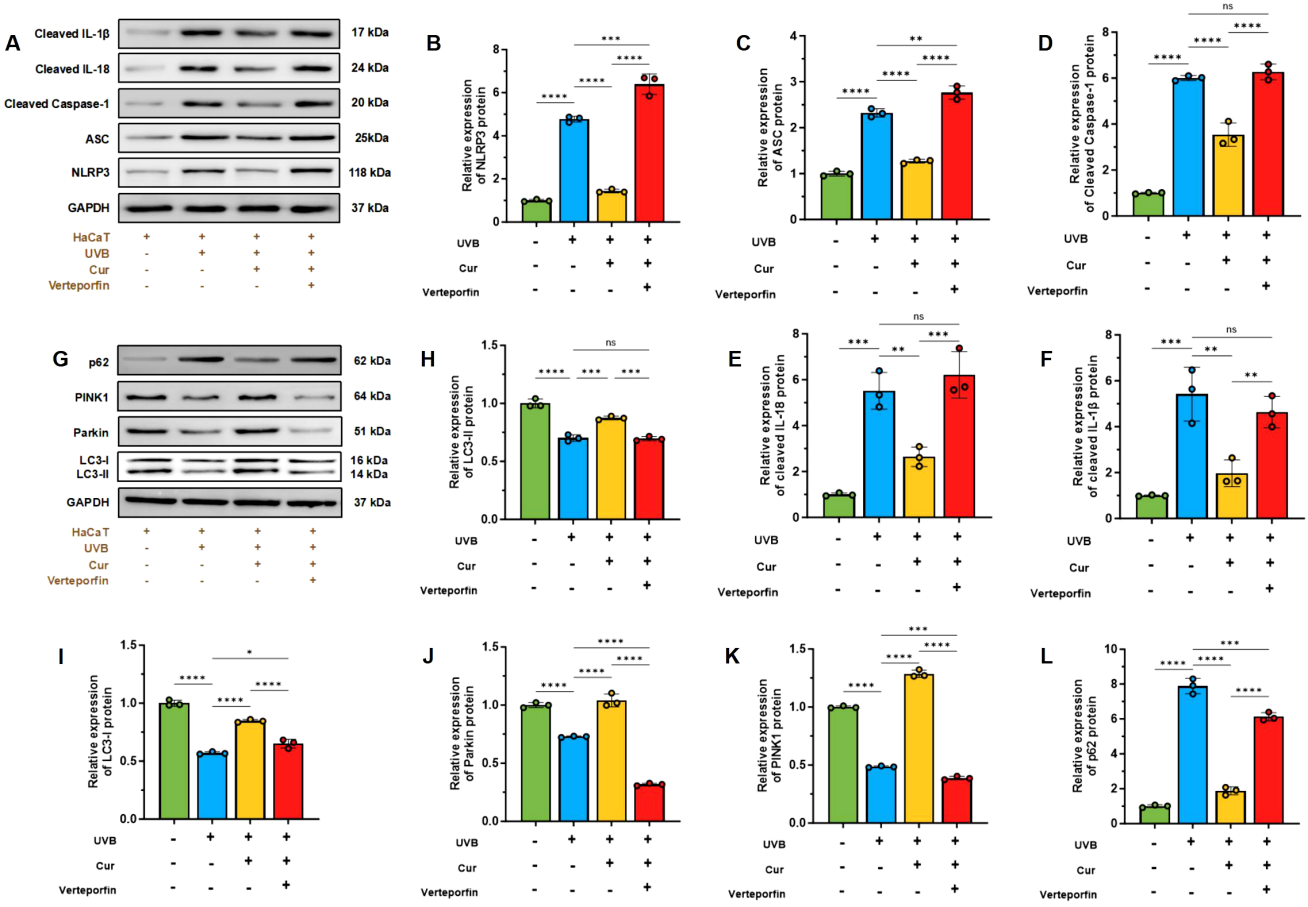
**(A)** Western blot analysis of NLRP3, ASC, cleaved Caspase-1, IL-18, and IL-1β. **(B–F)** Relative expression of NLRP3, ASC, Cleaved Caspase-1, IL-18, and IL-1β (n=3). **(G)** Western blot analysis of P62, PINK1, Parkin, LC3-I, and LC3-II. **(H–L)** Relative expression of P62, PINK1, Parkin, LC3-I, and LC3-II (n=3). The data are presented as mean value ± SD. Statistical significance was calculated using one-way analysis of variance (ANOVA) with Tukey multiple comparison. ns *P* > 0.05, * *P* < 0.05, ** *P* < 0.01, *** *P* < 0.001, **** *P* < 0.0001.

The expression levels of key molecules in the IL-18 signaling pathway within HaCaT cells were assessed to elucidate the effects of UVB radiation and curcumin treatment. Our findings indicate that UVB exposure activates the IL-18 pathway, as evidenced by the upregulation of NLRP3, ASC, Cleaved Caspase-1, IL-18, and IL-1β. Comparatively, the expression levels of these molecules were reduced in the UVB+curcumin treatment group (**Figures 7A–F**).

Furthermore, we conducted a WB analysis to evaluate the expression of mitochondrial autophagy-related proteins in HaCaT cells, thereby assessing the protective role of curcumin against UVB-induced mitochondrial damage. The results demonstrated that UVB radiation alters the expression levels of PINK1, Parkin, the LC3-II/LC3-I ratio, and P62. Curcumin intervention elevated the LC3-II/LC3-I and PINK1/Parkin ratios. Additionally, UVB increased the expression of P62, which was decreased by curcumin treatment. However, the addition of the YAP1 inhibitor Verteporfin led to a decrease in the LC3-II/LC3-I and PINK1/Parkin ratios and an increase in P62 expression (**Figures 7G–L**).

## 4 Discussion

This study employed a comprehensive experimental design, encompassing both an *in vivo* mouse UVB irradiation model and an *in vitro* HaCaT cell UVB exposure model, to investigate the protective effects of curcumin on UVB-induced skin damage and its underlying mechanisms. The study presents a comprehensive analysis of the protective effects of curcumin against UVB-induced skin damage, as demonstrated through a series of *in vivo* and *in vitro* experiments. The findings provide a detailed understanding of the multifaceted protective mechanisms of curcumin against the adverse effects of UVB exposure. The *in vivo* experiments using a UVB-induced skin damage mouse model revealed that curcumin treatment ameliorated skin damage, as evidenced by reduced severity scores and improved histological parameters. The dose-dependent protective effect of curcumin was further substantiated by the restoration of skin architecture and a decrease in inflammatory cell infiltration. This is complemented by the reduction in epidermal and dermal thickness, which was increased by UVB and normalized by curcumin treatment, particularly at higher concentrations.

Molecular analysis of the YAP1 signaling pathway, a key mediator of cell survival and proliferation, was a focal point of this study. Western blot analysis revealed that curcumin not only reduced the expression of pro-apoptotic markers but also enhanced the anti-apoptotic Bcl-2, suggesting a pro-survival effect. The scratch assay results, showing an improvement in cell migration rates post-curcumin treatment, further support its role in cellular repair and regeneration. The direct interaction between curcumin and YAP1 was explored using co-treatment with the YAP1 inhibitor Verteporfin, which abrogated the protective effects of curcumin. This suggests that the activation of the YAP1 pathway is integral to curcumin’s mechanism of action. The findings from the RNA sequencing analysis, which revealed an upregulation of YAP signaling pathway genes post-curcumin treatment, align with these results and provide a molecular basis for curcumin’s therapeutic effects. *In vitro* experiments further substantiated these findings by demonstrating curcumin’s ability to counteract UVB-induced oxidative stress, as measured by the DCFH-DA assay, and to modulate mitochondrial health, as indicated by the JC-1 assay. Curcumin’s role in promoting mitochondrial autophagy was highlighted by the increased LC3-II/LC3-I ratio and decreased expression of P62, a marker of mitochondrial damage.

In conclusion, curcumin emerges as a promising therapeutic agent for UVB-induced skin damage, exerting its effects through the modulation of the YAP1 signaling pathway, enhancement of mitochondrial autophagy, and suppression of inflammatory responses. These findings pave the way for future research into the clinical application of curcumin in photodermatological conditions and highlight the importance of targeting the YAP1 pathway in the development of novel therapeutic strategies for skin protection and repair. The discovery of curcumin’s mechanism of action provides a foundation for the development of new therapeutic approaches that could potentially be translated into clinical practice, offering a new avenue for the treatment of UVB-induced skin damage.

However, this study has certain limitations, such as mainly focusing on UVB-induced skin damage and insufficient research on skin damage caused by UVA and other environmental factors. In addition, although curcumin has shown significant protective effects, further research is needed to fully understand its mechanism of action, especially its interaction with other signaling pathways. Future research should further explore the applicability of curcumin in different skin types and ethnic populations, as well as its safety and efficacy in clinical applications. In addition, developing new synthetic strategies to enhance the bioavailability and stability of curcumin, as well as exploring its potential applications in other inflammatory diseases, are also important directions for future research.

## Data Availability

The original contributions presented in the study are included in the article/**Supplementary Material**. Further inquiries can be directed to the corresponding author.
