# Supplementary figures and images for: Curcumin targets YAP1 to enhance mitochondrial function and autophagy, protecting against UVB-induced photodamage
Source: Front Immunol. 2025 Mar 25;16:1566287. doi: 10.3389/fimmu.2025.1566287 (PMC11975583; doi:10.3389/fimmu.2025.1566287)

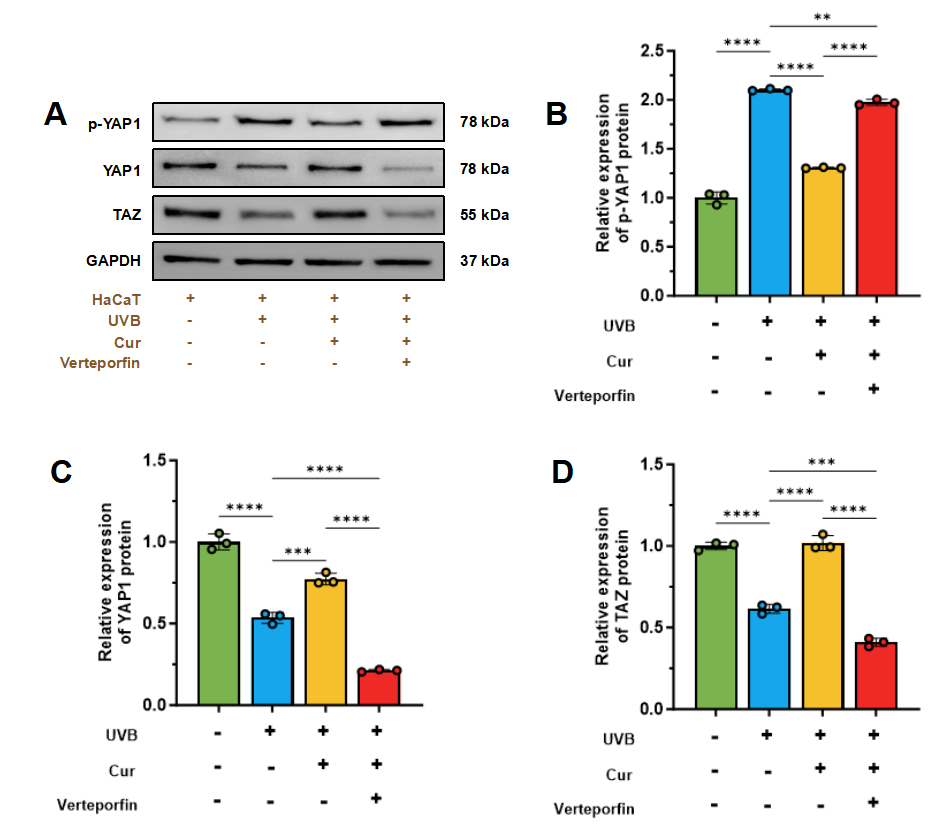

Supplement: Supplementary file 1 [file Image1.tif]

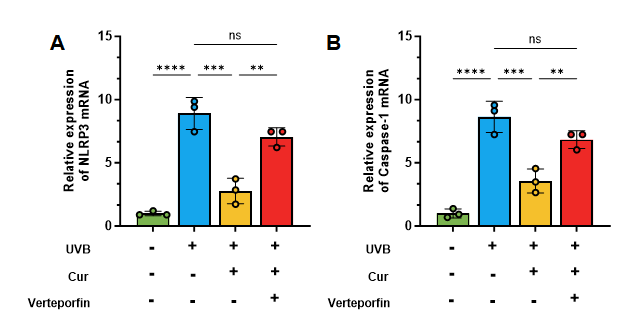

Supplement: Supplementary file 2 [file Image2.tif]

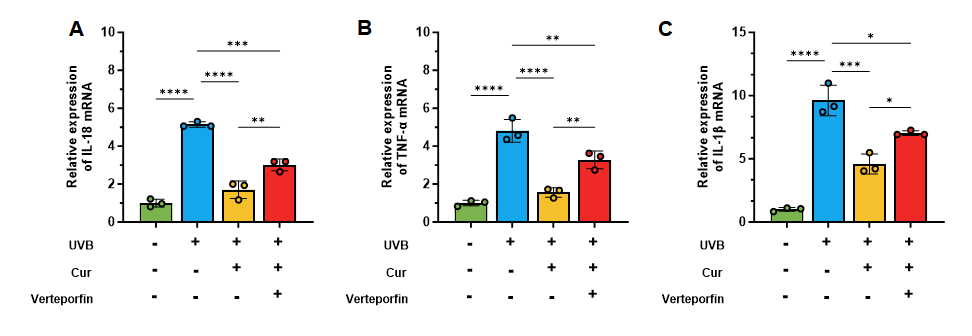

Supplement: Supplementary file 3 [file Image3.tif]
